# Conceptual design of a decision knowledge service model integrating a multi-agent supply relationship diagram for electric power emergency equipment

**DOI:** 10.3389/fdata.2025.1603106

**Published:** 2025-06-06

**Authors:** Jiandong Si, Chang Liu, Jingxian Ye, Jianfeng Wu, Jianguo Wang, Kairui Hu, Chunhua Ju, Qianwen Cao

**Affiliations:** ^1^State Grid Jinhua Power Supply Company, Jinhua, Zhejiang, China; ^2^Modern Business Research Center, Zhejiang Gongshang University, Hangzhou, Zhejiang, China; ^3^College of Artificial Intelligence & E Commerce, Zhejiang Gongshang University Hangzhou College of Commerce, Hangzhou, Zhejiang, China

**Keywords:** electric power emergency supplies, relationship diagram, supply decision, intelligent optimization, conceptual design

## Abstract

**Introduction:**

The decision regarding the supply of emergency equipments for power emergencies requires timeliness, efficiency, and accuracy. The multi-agent supply relationship graph, based on complex data fusion, enables the comprehensive exploration of interconnections among key entities in power emergency supplies.

**Methods:**

This approach enhances decision-making efficiency and quality by uncovering multiple relationships between main bodies involved. The present study focuses on the decision-making process for power emergency equipments supply and aims to enhance its professionalization. To achieve this goal, multi-modal data regarding power emergency equipments supply is collected from both internal and external power enterprises. Subsequently, a decision support knowledge base is established, along with a four-dimensional relationship graph that integrates events, time, equipments, and suppliers based on the knowledge graph. This enables the mining of multidimensional relationships pertaining to the main body. Finally, supported by the graph, the platform can offer intelligent assistance in decision-making, supplier recommendation, optimization of emergency equipment scheduling for electric power supply, and provides effective information and guidance for decision-making in electric power emergency equipment supply.

**Results:**

After conducting a comparative analysis, the decision support system based on the knowledge graph proposed in this study demonstrates superior effectiveness and precision. By integrating the four-dimensional relationship graph with data mining algorithms, precise decision support can be provided for power emergency response. After verification through case studies, the model developed in this study was utilized to recommend suppliers of power emergency equipment, and the recommendation results demonstrated a closer alignment with actual procurement outcomes.

**Conclusion and recommendation:**

This system proposed by this study delivers multidimensional knowledge guidance and optimized decision pathways for emergency supply management.

## 1 Introduction

Effective management of electric power emergencies is critical for preventing, mitigating, and minimizing the impact of unforeseen disruptions. In recent years, frequent natural disasters have triggered widespread power outages, severely affecting both livelihoods and industrial production. Rapid restoration of power supply during emergencies is essential to minimize socioeconomic disruptions. Moreover, given the inherent unpredictability of large-scale power outages, a robust emergency equipment dispatching and response system is indispensable for effective crisis management. Such a system enables data-driven decision-making for emergency resource allocation, thereby reducing losses during power crises. The integration of digital technologies and big data analytics enables knowledge-based decision-making for power emergency supply management, providing scientifically grounded, rational, and efficient allocation strategies. Furthermore, this approach significantly reduces costs and improves energy efficiency, positioning it as the future standard for power emergency supply decision-making.

A knowledge graph (KG) is an intelligent knowledge base integrating principles from knowledge engineering, artificial intelligence, and traditional databases. KGs organize heterogeneous data while supporting intelligent information management, exploration, and mining (Wang et al., 2020). Furthermore, KGs facilitate intelligent search, question-answering systems, recommendation mechanisms, and decision-making support (Sawant et al., 2019; Li et al., 2021). These capabilities have seen growing adoption across finance, public security, healthcare, and other sectors (Zhu et al., 2024). The power industry has similarly adopted KG technologies for specialized applications including grid scheduling, marketing, operations, and equipment inspection (Sun et al., 2023; Zhao et al., 2015). KG construction typically involves four core processes: knowledge extraction, representation learning, knowledge mining, and knowledge reasoning/fusion (Wu et al., 2018).

Artificial intelligence technology facilitates knowledge graph construction by extracting nodes and relationships from extensive power system data, integrating complex information into a unified structure that enhances management efficiency (Wu et al., 2024). The processed data enables the establishment of power industry standards and offers practical guidance for engineering applications. KGs enable synchronization and coordination across power system subsystems by establishing relationships among system entities. Time-series-based dynamic knowledge graphs support real-time updates of nodes and relationships, enabling adaptation to both system upgrades and external environmental changes (Ji et al., 2021).

Platform-based organizations have gained widespread adoption across industries, enabling intelligent service integration, cross-departmental data sharing, and comprehensive service support. The platform model has become predominant in business, with Amazon and Alibaba representing prominent examples. O'Reilly extended the platform concept to government governance through the “government as a platform” paradigm, accelerating public administration digital transformation.

As demonstrated, KGs enable data-driven decision-making by systematically analyzing entity relationships, improving both operational efficiency and analytical accuracy through existing data resources. However, existing research lacks a comprehensive theoretical framework for integrating entity-relationship graphs with AI technologies using platform-based approaches. In practice, emergency power resource allocation requires consideration of multiple complex factors: resource inventories, multi-agent supply networks, socioeconomic conditions, and environmental variables. Systematically enhancing knowledge graphs is essential to bridge the supply-demand gap for emergency power resources. Thus, developing a multi-agent supply-relationship knowledge graph system for power distribution decision-making is critically important.

This study addresses these theoretical and practical challenges through two key research objectives:

(1) Developing an integrated theoretical framework combining inter-agent network relationships with platform organization theory, enhanced by multi-agent power supply relationship knowledge services to expand both domains' theoretical and applied boundaries.
(2) Creating a four-dimensional knowledge graph (events-time-equipments-suppliers) and corresponding decision-support platform to optimize intelligent power equipment supply decisions through multi-agent relationship modeling.

## 2 Literature review

### 2.1 The application of KG in electric power

Network structures effectively model complex relationships and serve as fundamental components in computational social science research (Lazer et al., 2020). Recent research has successfully applied network analysis across multiple domains, including finance (Kejriwal, 2021), crisis informatics (Sadri et al., 2021), and biotechnology (Szklarczyk et al., 2021). Specific network algorithms, particularly link prediction and community detection methods, have enabled advanced applications like collaborative forecasting and group pattern mining (Kumar et al., 2020; Jin et al., 2023; Ju and Cao, 2025).

KGs are built upon structured semantic knowledge bases that represent entities as nodes and relationships as edges in graph structures (Yan et al., 2018). The KG represents entities and relations as triplets, specifically in the form of “entity-relation-entity”, while the characteristics of entities are represented using the format of “attribute-attribute value” (Hogan et al., 2021). The construction process of a KG involves four essential steps: knowledge extraction, learning of knowledge representation, mining of knowledge, and reasoning and fusion of knowledge. The extraction of terms can be facilitated through the utilization of dictionaries, rules, statistics, and machine learning techniques (Etzioni et al., 2008). Relationship and concept extraction utilizes both linguistic and statistical methods (Zhou et al., 2014; Fu et al., 2020). Representation learning transforms real-world knowledge into computational representations (Viloria and Lezama, 2019). Traditional representation learning models include TransE, TransR, TransD, TransG, and TransH (Cesar et al., 2019). Recent advances have introduced new models including MGTransE (Warren et al., 2019) and KG2E (Lei et al., 2020). Knowledge mining identifies novel entity relationships to enrich the KG (Zhang et al., 2021). This phase utilizes link prediction, neural networks, and decision trees to infer implicit relationships. The goal is to discover potential future collaborative relationships. Finally, KGs require continuous updating to reflect evolving entities and relationships. Key updating algorithms include NBFNET (Zhu et al., 2021), PALT (Shen et al., 2022), KGLM (Youn and Tagkopoulos, 2022), and LP-BERT (Li et al., 2023).

An event relationship graph represents event logic through nodes (events) and directed edges (causal/consequent relationships), forming a cyclic structure that captures event development patterns and evolutionary mechanisms. As an extension of KGs, event graphs specifically model dynamic event causality and underlying mechanisms. Neural networks and pre-trained models now dominate event extraction tasks (Du and Cardie, 2020; Ma et al., 2022), outperforming traditional pattern matching and machine learning approaches. Relational recognition identifies and classifies textual relationship pairs, employing either rule-based knowledge bases (Kosari et al., 2024) or deep learning methods for implicit semantic relationship extraction (Che et al., 2021).

Relational recognition aims to identify relationship pairs in text and classify their semantic types. KGs enable semantic search and intelligent question answering by inferring implicit relationships from existing entity connections. This approach improves both search accuracy and result predictability. Furthermore, it supports abnormal behavior detection (Hatirnaz et al., 2020), managerial relationship analysis (Chen et al., 2020), and news article clustering (Kallipolitis et al., 2012). Intelligent Q&A systems enhance online customer service through advanced AI capabilities. Multi-modal Q&A systems have attracted considerable research attention. These systems have been applied in diverse domains including education (Yang et al., 2021), healthcare (Peng et al., 2025), voice interfaces (Kumar et al., 2017), tourism (Do et al., 2021), traditional medicine (Yu et al., 2017; Xiong et al., 2021), and finance (Ma et al., 2021).

KGs utilize existing node relationships to predict future connections. Consequently, they have become increasingly sophisticated with AI advancements, finding wide application in power forecasting, scheduling, and related domains. Current applications demonstrate significant efficacy in: power load forecasting (Yin and Xie, 2021; Sheng et al., 2021), fault diagnosis (Zera and Ayati, 2021), system optimization (Xi et al., 2021), resource scheduling (Ong et al., 2021), and maintenance detection (Liu et al., 2022).

### 2.2 Power emergency equipments supply decision-making

Emergency power equipment distribution utilizes demand forecasting and storage facility locations to enable precise dispatching, ensuring supply-demand matching throughout the process and effectively reducing the impact of power outages. Non-standardized emergency power equipments storage complicates reserve quantity determination and increases response time requirements.

Effective reserve quota management ensures adequate power grid emergency equipment reserves, while analyzing key influencing factors is essential for quota optimization (Huang, 2019). Scholars have applied the DEMATEL method to quantify interrelationships among indicators and classify power emergency equipments by identifying key influencing factors (Zhang et al., 2024). For reserve warehouse location, researchers have developed the fuzzy evaluation model for site selection, multi-objective path optimization model (Wan et al., 2023), and the mobile storage configuration model (Jiang et al., 2021). For equipment distribution, Mojtaba et al. (2016) developed a dynamic model maximizing disaster site utility under supply-demand, time, and transportation constraints. Zhuang and Zhang (2023) utilized the SEIR model to predict emergency equipment demand during public health crises and also created an optimization model minimizing both shortage impacts and distribution distances.

### 2.3 Limitations of current research

Current research on knowledge graphs, relationship graphs, and power emergency supply decision-making has developed effective optimization methods and produced significant findings in supply strategies. However, scholars have largely overlooked the relationship between graph construction and electric emergency supply management. Optimizing power emergency supplies requires intelligent, data-driven decision-making with scientific rigor and timeliness. Rational allocation and scientific planning are essential for multi-agent supply chain coordination. Relationship graphs effectively model entity relationships and demonstrate strong event reasoning capabilities. Current knowledge and reasoning graphs fail to adequately model the complex time-event-supplier-equipment relationships in power emergency supply scenarios. They also lack comprehensive decision-support capabilities for emergency supply management. Therefore, a multi-agent supply relationship graph system would provide significant theoretical and practical benefits for power emergency decision-making. This approach would both expand knowledge graph applications and optimize power emergency supply decisions.

## 3 Methods

Following platform organization principles, developing a decision knowledge service system involves three core tasks: (1) Role integration within the system; (2) Platform module design using reusable knowledge bases and algorithm libraries; (3) Coordinated resource integration among stakeholders including equipment suppliers, power departments, technical personnel, and management entities. This internet-connected, multi-terminal platform enables efficient emergency equipment management and intelligent decision support.

**Figure 1:**
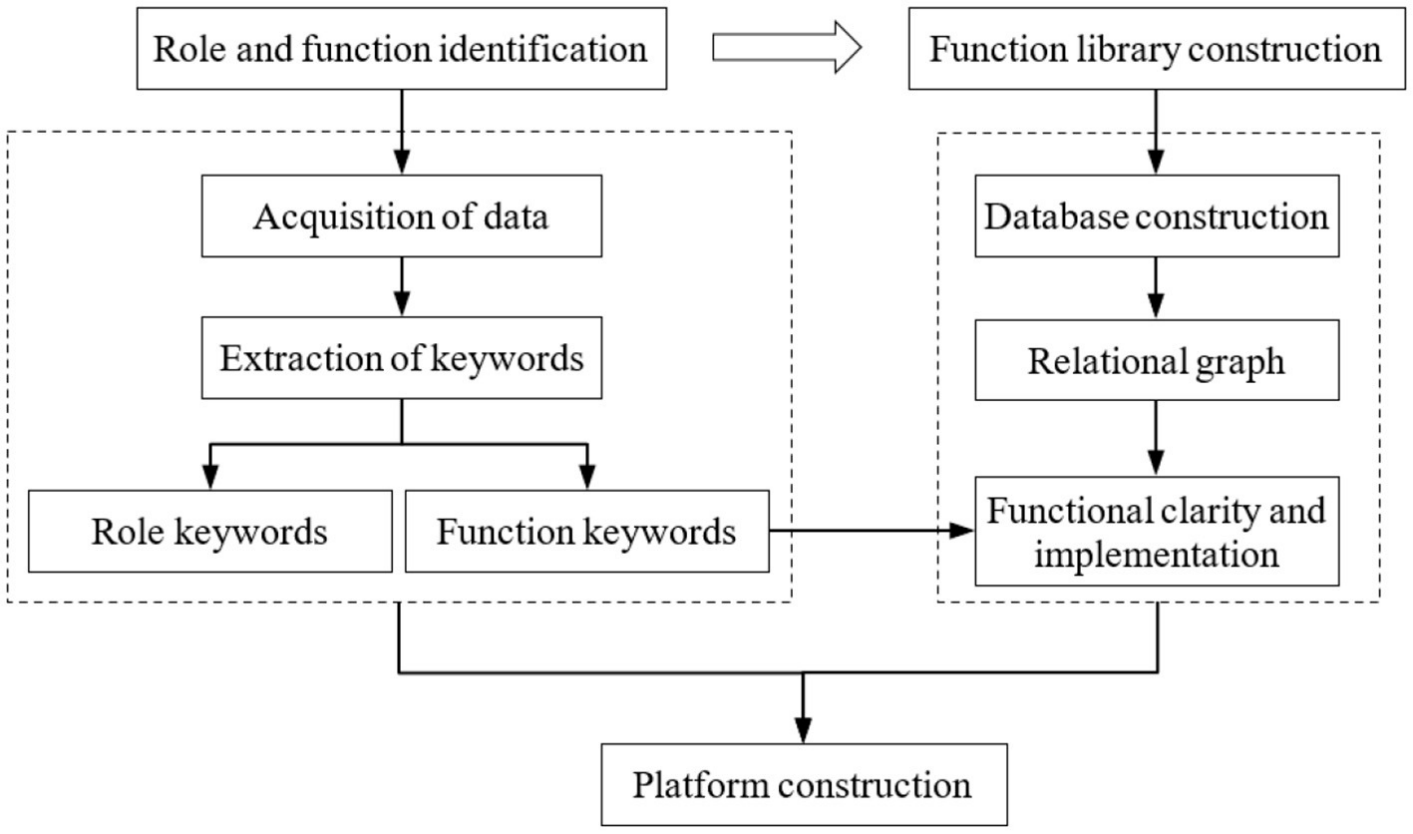
The method of platform construction.

This study follows a three-platform framework construction process: (1) Role and function identification, (2) function library construction, and (3) platform construction, as shown in Figure 1.

### 3.1 Roles and functional identification

Big data-driven role identification for power emergency equipment decision-support platforms, augmented by process expertise, improves operational accuracy. Domain policy documents provide comprehensive guidelines that govern all roles and behaviors within the power emergency ecosystem. Keyword extraction from these policy documents identifies role-specific functions, clarifying the platform's operational responsibilities.

First, we collected power emergency equipment supply policies from the Chinese government's official website to build the text corpus. Second, we used Python's Jieba library for Chinese word segmentation, followed by stop-word removal and TF-IDF analysis to identify term importance. Finally, we ranked roles and functions by TF-IDF values, selecting the top five most significant contributors for platform development.

### 3.2 Function library construction

#### 3.2.1 Database construction

Following role identification, we acquire data and construct preliminary databases through two primary approaches: (1) Role-specific internal data including workflows and equipment metadata; (2) Open data including patents and standards which involve structured, semi-structured, and unstructured data formats. Comprehensive data collection is essential for optimal platform functionality.

#### 3.2.2 Relational graph construction

##### 3.2.2.1 Entity/characteristic extraction

Entity/characteristic extraction identifies and extracts relevant terms from power emergency-related unstructured data. We employ a Hidden Markov Model (HMM) for corpus classification. In HMM-based segmentation, each observed word corresponds to a hidden state. The state set G = {B, M, E, S} represents: B-beginning, M-middle, E-end, and S-single character words. We augmented segmentation accuracy using a domain-specific power emergency terminology dictionary. Segmented words matching the domain dictionary become knowledge graph entity/characteristic nodes.

##### 3.2.2.2 Coreference resolution

The term “coreference resolution” refers to the process of identifying and linking entity/characteristic that have the same meaning but are expressed differently within the power emergency equipments corpus. The specific steps are as follows: (1) Tokenization using Python's Jieba package and POS tagging; (2) The word2vec algorithm is employed to vectorize each term within the set of parts of speech. The vector dimension is set to 50, and the part of speech set after vectorization is V_word_ = (v_1_, v_2_, v_3_, L, v_50_). (3) Similarity calculation via Formula 1 (higher values indicate stronger semantic similarity). Coreference established at cosθ ≥ 0.88 threshold.


(1) $$\begin{array}{l} cos\theta =\;\frac{x_{1}\cdot y_{1}+x_{2}\cdot y_{2}+x_{3}\cdot y_{3}+L+x_{50}\cdot y_{50}}{||\sqrt{x_{1}^{2}+x_{2}^{2}{+x}_{3}^{2}+L+x_{50}^{2}}||\cdot ||\sqrt{y_{1}^{2}+y_{2}^{2}{+y}_{3}^{2}+L+y_{50}^{2}}||} \end{array}$$

##### 3.2.2.3 Relationship extraction

**Figure 2:**
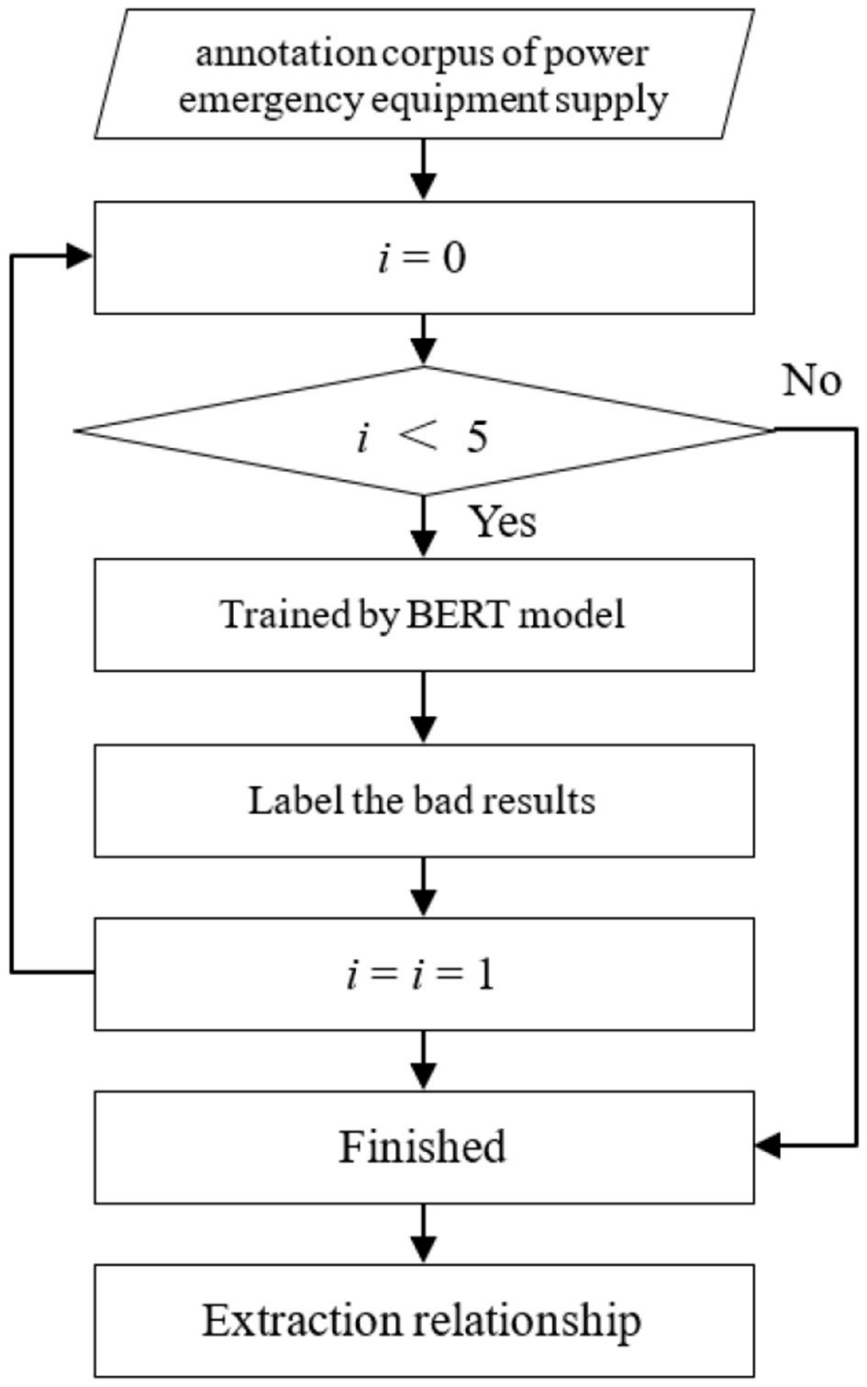
Relationship extraction process.

Relation extraction identifies relationships between entities in power emergency equipment corpora. We employ BERT for domain-specific relationship extraction due to its effectiveness with power systems' unique attributes. Figure 2 illustrates the relationship extraction pipeline.

##### 3.2.2.4 4-dimensional relationship construction

Traditional knowledge graphs represent facts as triplets (v1, r, v_2_), where v1 and v_2_ are entities connected by relation r. However, power emergency supply decisions require more comprehensive relationship representations than single triplets provide. They demand multi-dimensional relationship characterization. We propose a four-dimensional graph architecture for power emergency supply decision support, incorporating: (1) knowledge domains, (2) top-level designs, (3) spatiotemporal sequences, and (4) equipment responses, enabling efficient knowledge reasoning.

**Figure 3:**
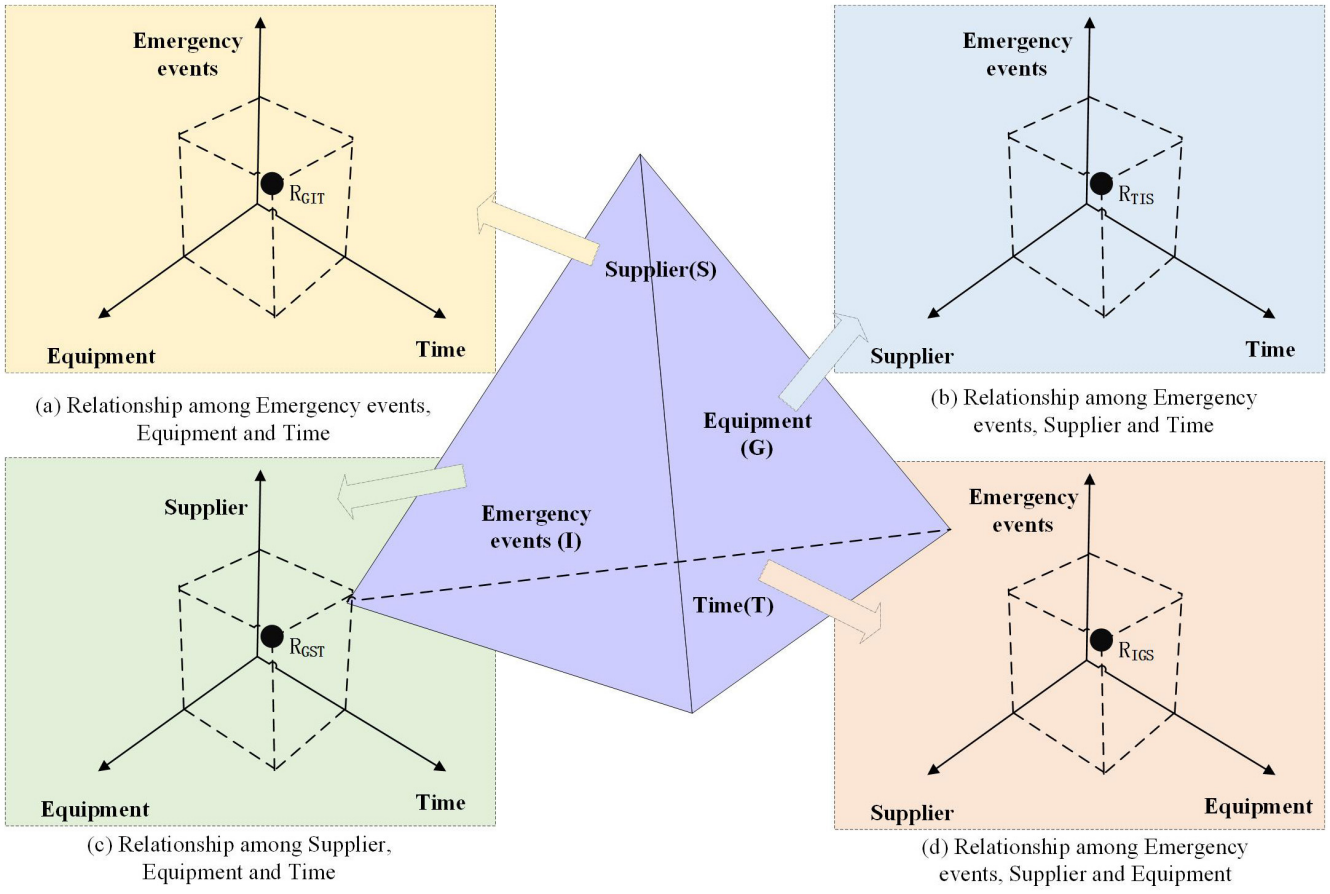
Construction logic for the four-dimensional relationship graph of power emergency supply.

As shown in Figure 3, our four-dimensional relationship graph is formally defined as Gr = (S, G, I, T, R), where:

S: supplier dimension (entity and attributes)

G: equipment dimension (required resources and attributes)

I: event dimension (emergency characteristics)

T: temporal dimension (event timeline)

R: relationship set (inter-dimensional connections)

R = {R (S, G), R (S, I), R (S, T), R (G, I), R (G, T), R (I, T), R (G, I, T), R (S, I, T), R (S, G, T), R (S, G, I)},

The relation set R contains logical reasoning relationships such as conditional, compositional, and causal relations.

Figure 3a depicts event-time-equipment relationships centered on emergency equipment suppliers. It shows temporal event patterns and corresponding equipment supplies. Figure 3b illustrates, with suppliers as the focal nodes, the provider-equipment correlations during power emergency events. Figure 3c analyzes supplier-equipment-time responses to emergency events. Figure 3d visualizes event-supplier-equipment temporal interactions at discrete time points. Each graph's vertices represent pairwise entity correlations. These 4D relationships enable historical and cross-event comparative analyses. Reasoning techniques applied to these models yield scientific decision support for emergency supply management.

### 3.3 Function clarity and platform construction

Platform functions are determined through combined function keyword extraction and expert consultation, guiding appropriate data mining technique selection. These functional requirements were then implemented using the relational graph structure. The platform architecture provides accessible, encapsulated function packages, maintaining core platform principles while ensuring scalability. The final architecture incorporates specialized functionalities for power emergency equipment decision support.

## 4 Conceptual design of a decision-knowledge service model

### 4.1 Data sources

We collected 23 highly relevant policy documents (2014-present) from Chinese government portals, selected through role-function analysis. For relational graph construction, we utilized emergency equipment management data from State Grid's Jinhua branch. This dataset reflects years of accumulated emergency response experience. We anonymized data by replacing organizational identifiers with numerical codes. Preprocessing included: (1) missing value imputation, (2) duplicate removal, and (3) noise filtration. The dataset contains both structured and unstructured records: (1) Structured: equipment inventories, demand logs (standardized formats); and (2) Unstructured: incident reports (variable formats requiring complex processing).

### 4.2 Role identification and function identification

Applying the Section 3.1 methodology, we extracted keywords, computed TF-IDF values, and generated separate rankings for role and function keywords. Table 1 presents these keyword rankings.

**Table 1:** Roles and functions of knowledge service platform.

**Role keywords**	**TF-IDF**	**Function keywords**	**TF-IDF**
State Grid	0.0946	Supply	0.1121
Electricity user	0.0766	The plan	0.0997
Supplier	0.0631	Protection	0.0981
Company of equipments	0.0541	Scheduling	0.0659
Power substation	0.0482	Early warning	0.0535

### 4.3 Function library construction

**Figure 4:**
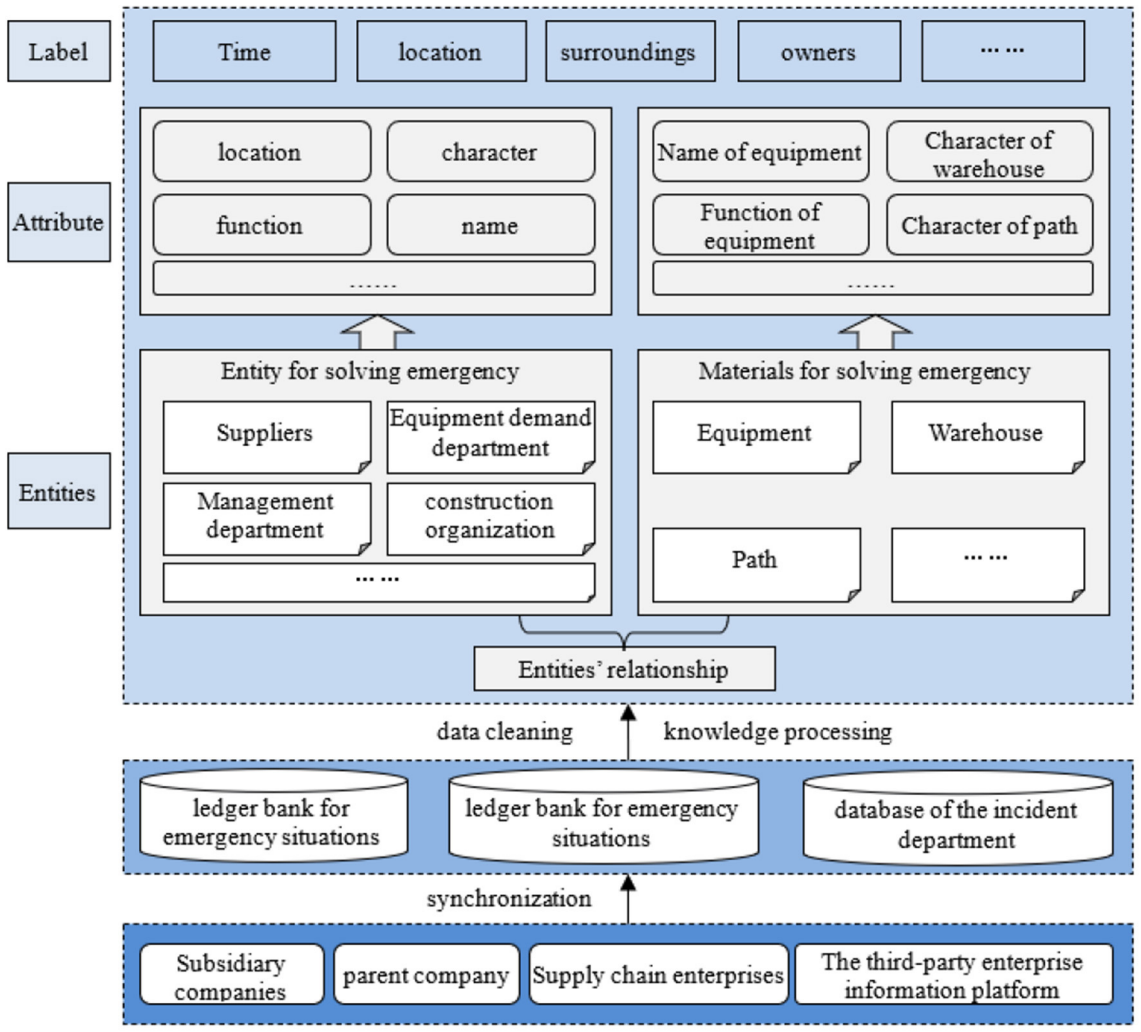
The construction process of the knowledge base for power emergency supplies supply.

Constructing the power emergency supplies knowledge base requires four key processes: (1) information acquisition, (2) knowledge extraction, (3) knowledge fusion, and (4) knowledge updating. Figure 4 details this knowledge base construction workflow.

#### 4.3.1 Acquisition and classification of knowledge sources

**Figure 5:**
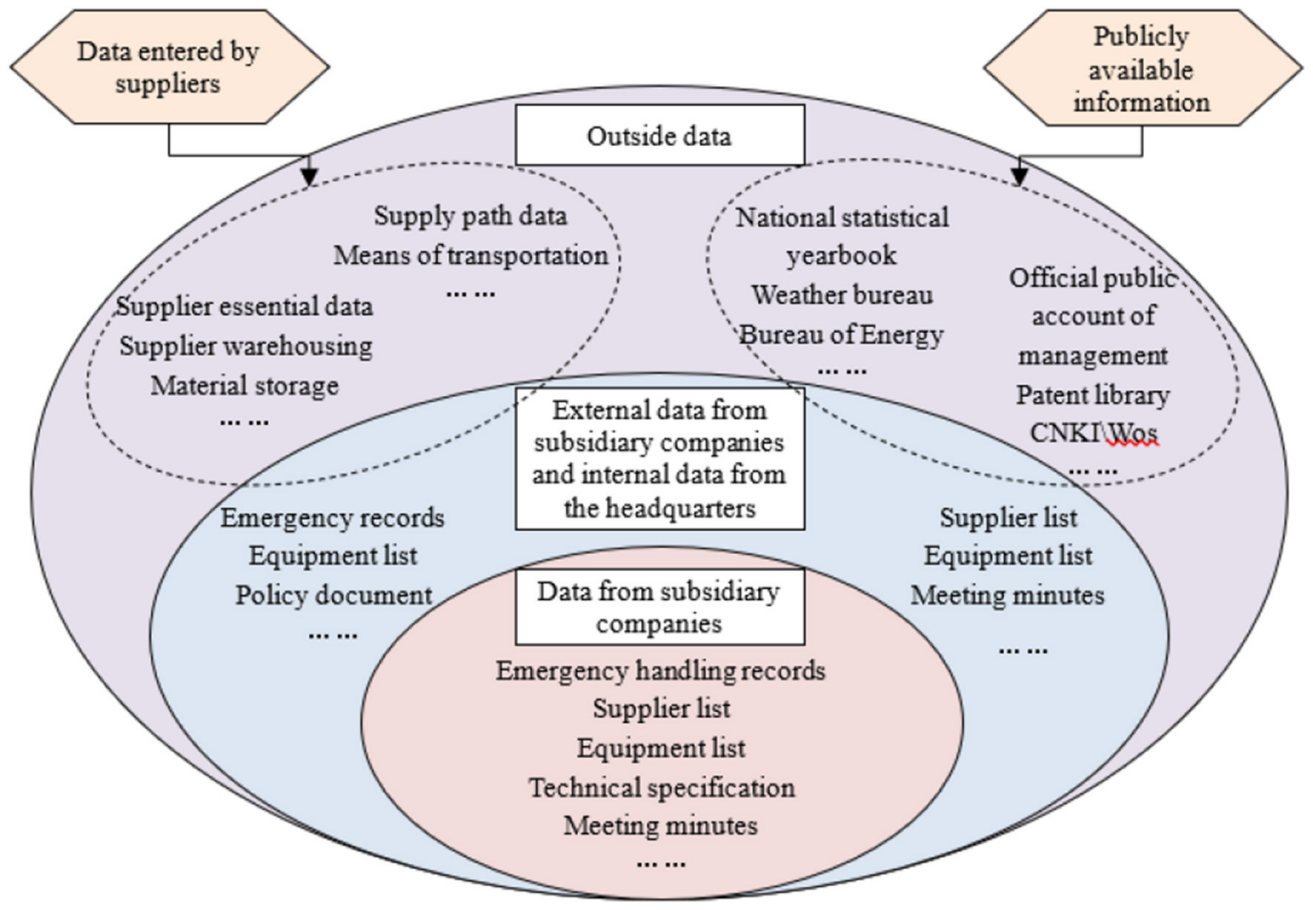
Data source and classification.

Building a reliable knowledge service platform for power emergency supplies requires verifiable, objective data sources. These sources form the foundation for modeling supply-chain relationships in power emergency systems. The platform must integrate multi-format data including structured, semi-structured, and unstructured statistics, texts, and charts. The knowledge base must span multiple administrative levels, from international standards to local enterprise data, to adequately support emergency power supply needs. Power emergency supply information sources divide into internal (professional) and external categories. Internal sources include both subsidiary-level and cross-departmental headquarters data. External sources consist of supplier-provided data and publicly available information. Data integration leverages internal expertise while incorporating diverse external sources. Figure 5 details this knowledge source classification framework.

##### 4.3.1.1 Internal information

Internal data originates from power companies, comprising emergency event records, equipment inventories, supplier databases, and related operational data. Company intranets host additional headquarters documents including standards, notices, and minutes, along with provincial and subsidiary company equipments. Structured/semi-structured internal data includes technical specifications, feasibility studies, project acceptance reports, and internal media releases. These datasets require classification, organization, thematic tagging, and keyword/phrase extraction.

##### 4.3.1.2 External information

External data collection integrates multiple sources, including public online data and supplier submissions. Web-based sources provide economic indicators, policy documents, industry trends, and corporate performance metrics. Regulatory agencies' websites serve as primary sources for official industry development news. Energy corporations' websites offer operational updates through news releases and official statements. Power companies additionally publish industry-specific research reports through official channels. These include expert policy analyses and industry development interpretations. Scientific research data provides theoretical foundations for optimizing emergency power supply decisions. This research data originates from global academic platforms. Patent data is acquired from Derwent Innovation Index. Supplier data interfaces provide access to: business registrations, equipment specifications, storage capacities, and transportation logistics.

#### 4.3.2 Label definition

Entity labeling in the knowledge base enables efficient resource management while supporting entity relationship mapping and knowledge mining. Labels are metadata-defined, describing entity/attribute characteristics. This framework supports resource discovery, retrieval, management, and tracking. Our template-based metadata extraction analyzes power emergency resources from: (1) academic databases (e.g., CNKI), (2) government documents, and (3) energy company publications. Key metadata attributes encompass topics, keywords, publishers, timestamps, and usage metrics. The labeling system allows customizable field-specific collection, intelligent label-entity matching, and autonomous semantic expansion.

#### 4.3.3 Construction logic of entity relationship graph

##### 4.3.3.1 Ontology and relationship construction

**Figure 6:**
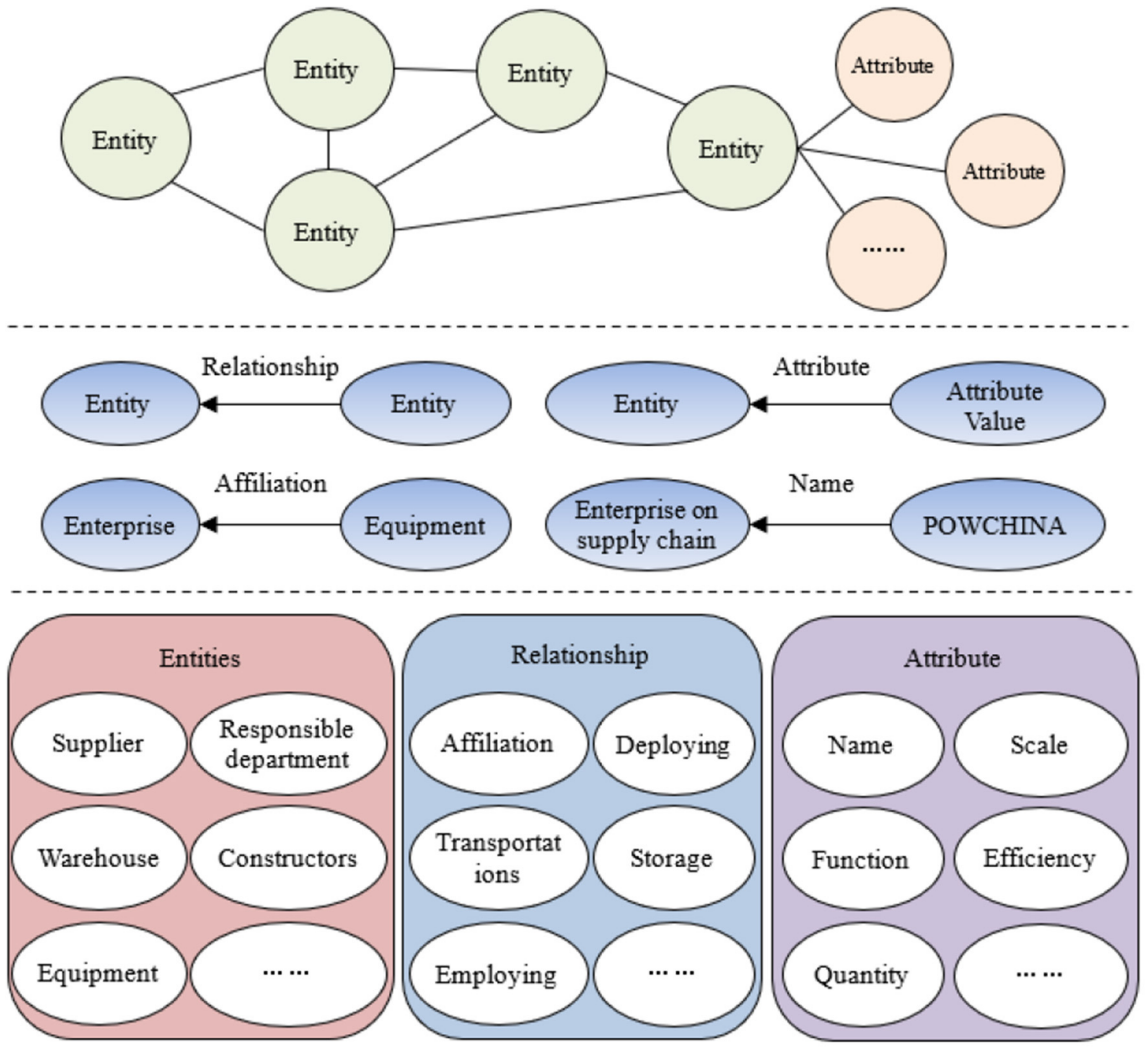
Power emergency equipment supply ontology and relationship construction.

Figure 6 illustrates the fact ontology construction process through data-to-model layer mapping. This transformation converts knowledge databases into structured graphs while supporting unified knowledge management.

The emergency power supply knowledge base integrates multidisciplinary data from diverse sources. Its hybrid methodology combines data-driven bottom-up processing and knowledge-driven top-down structuring. The bottom-up approach analyzes structural patterns to incrementally build ontologies from heterogeneous external data. The top-down method leverages existing power system knowledge to enhance KG robustness for complex scenarios.

**Figure 7:**
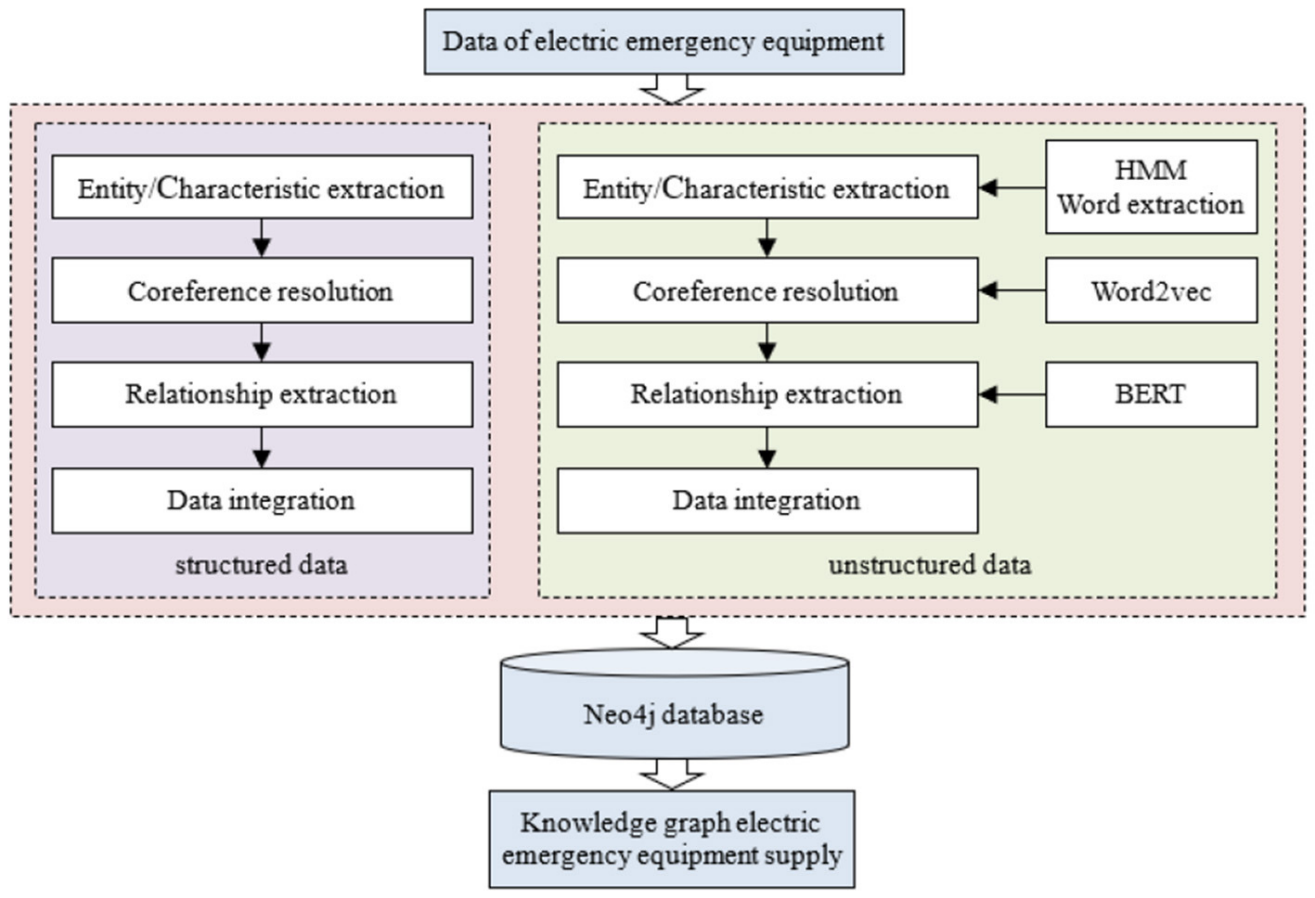
Workflow of data processing and graph construction.

While structured data imports directly into databases, unstructured data frequently contains input errors and semantic ambiguities from manual processing. Data cleansing ensures unstructured data accuracy. We first apply a Hidden Markov Model (HMM) for semantic analysis, then combine it with domain-specific terminology to perform word segmentation. Next, we filter irrelevant corpus using a power emergency equipment dictionary. Finally, we detect and remove anomalies in the emergency equipments data. Figure 7 details this workflow.

#### 4.3.4 Relationship extraction and visualization of relationship graph

**Figure 8:**
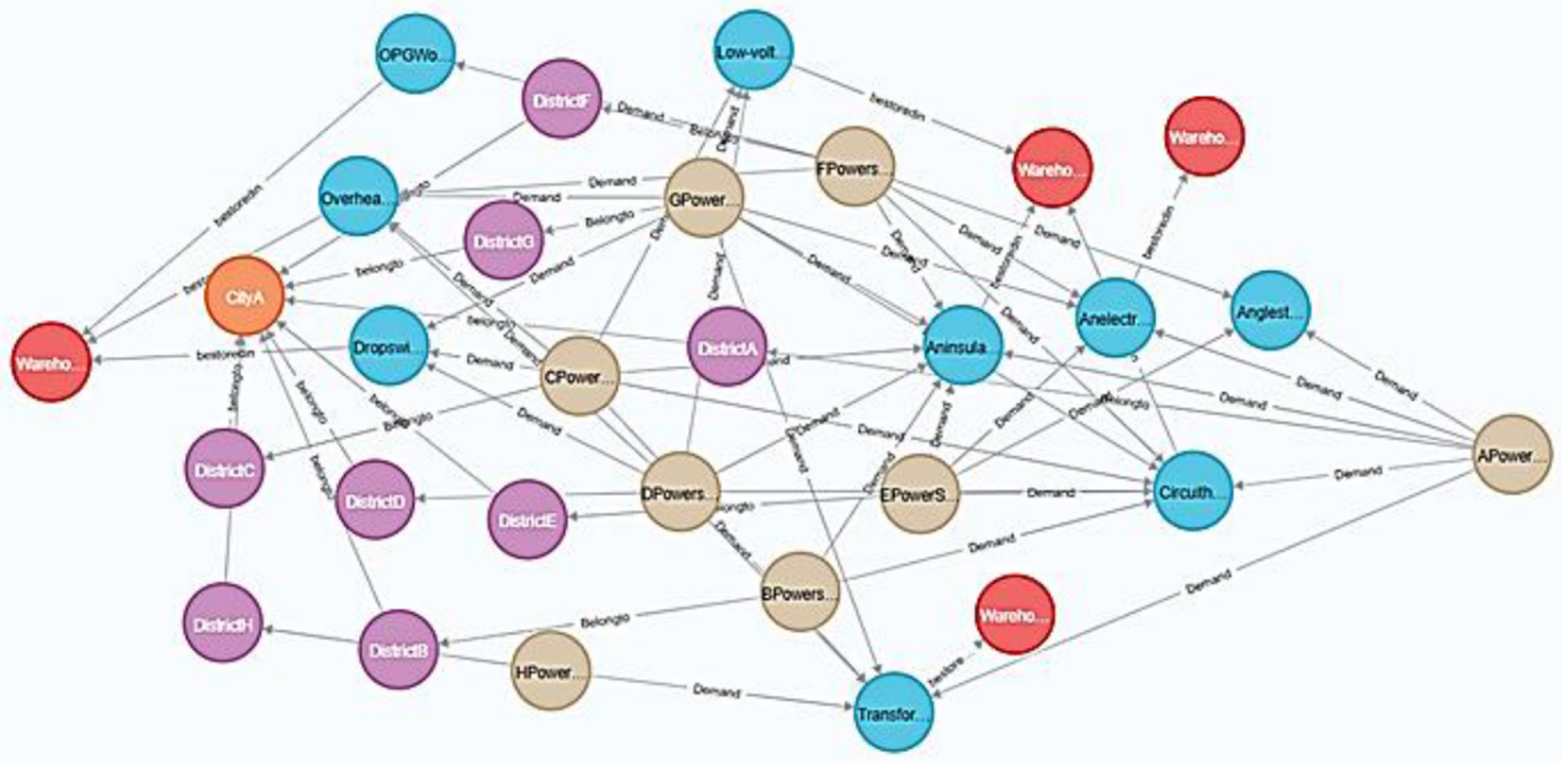
Relationship graph of electric power emergency equipments.

Using the Section 3.2.2 relation extraction method, we establish inter-entity relationships. This extraction yields multidimensional relationships (Figure 4) between identified entities. We implement these relationships in Neo4j, producing the relationship graph shown in Figure 8.

### 4.4 Platform architecture design

**Figure 9:**
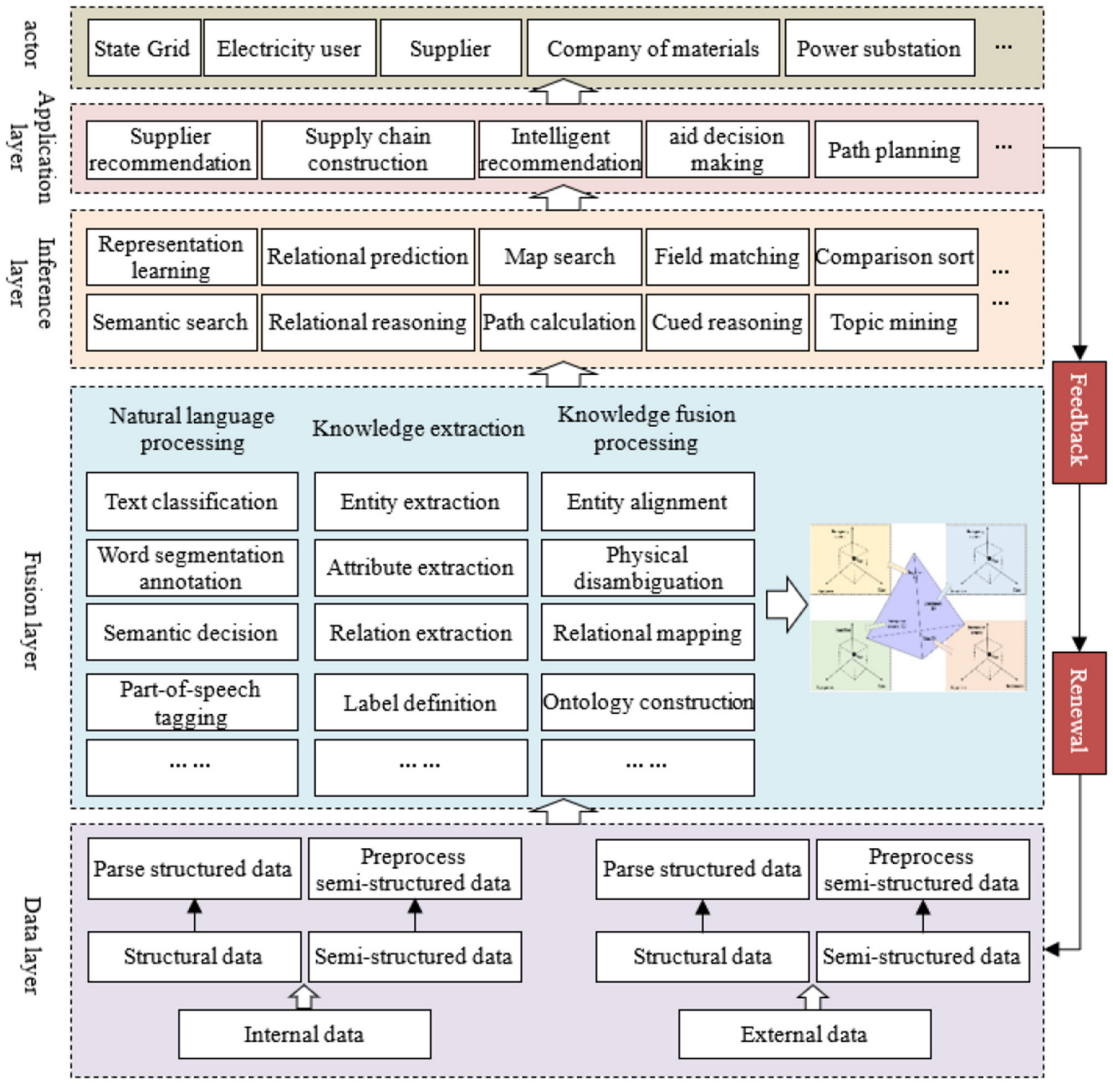
Functional architecture of the power emergency equipments supply decision support service platform.

The Power Emergency Equipments Decision-Support Platform is designed to meet operational requirements of emergency power supply management. By leveraging comprehensive supply chain data, it delivers intelligent decision-support capabilities for emergency response scenarios. Shown as Figure 9.

#### 4.4.1 Data layer

The data layer performs core data acquisition and preprocessing functions. Data sources, which contains predominantly semi-structured and unstructured documents and minimal structured data, include both internal emergency records and external inputs from suppliers, policies, patents, and literature. This layer acquires/stores raw data and analyzes structured formats (Excel, JSON, etc.) through import, parsing, and structured storage operations. Then semi-/unstructured data preprocessing includes text cleansing (noise removal, term normalization) and object standardization.

#### 4.4.2 Fusion layer

The fusion layer critically enables power emergency knowledge base construction. A multi-agent relationship map integrates fragmented data to support knowledge base development. This layer performs NLP, knowledge extraction, and integration functions. NLP focuses on text annotation through segmentation, semantic analysis, POS tagging, corpus classification, and topic modeling. These techniques enable deep knowledge understanding. Neo4j stores entities, attributes, and relationships within the fusion layer, which supports ontology fact creation, many-to-many relationship mapping and robust knowledge base development with advanced reasoning capabilities.

#### 4.4.3 Inference layer

The inference layer supplies algorithmic models for upper-level applications, including representation learning, relational inference, graph search, path computation and contextual inference. Representation learning converts inputs into discriminative features to interpret user intent. Relational inference deduces logically connected knowledge points from user queries. Graph search accurately matches queries to knowledge base elements. Path computation generates adaptive learning trajectories by optimizing knowledge sequences. Contextual inference analyzes trending topics and provides predictive insights.

#### 4.4.4 Application layer

As the platform's output module, the application layer enables user interaction and real-world implementation through supplier recommendation/valuation, intelligent supply chain management, decision support with path planning and emergency technology forecasting. Beyond operational decision-making, it provides research insights for power emergency management. Role-based access control with tiered portals and confidentiality protocols ensures enterprise-grade security.

#### 4.4.5 Result feedback

The platform incorporates a feedback mechanism that evaluates prediction accuracy during user interactions and overall system performance. Specific metrics assess supplier recommendation quality and emergency resolution efficacy. Feedback analysis drives continuous improvement through data/algorithm optimization, relationship graph reasoning updates and supply-demand pattern discovery - enabling precise decision support.

### 4.5 Validation of the decision knowledge service model

#### 4.5.1 Validation of model validity through recommendations for power emergency equipment suppliers

Our multi-agent power supply relationship platform provides intelligent support for emergency decisions, supplier recommendations and equipment scheduling optimization. Given system complexity, we focus validation on representative supplier recommendations. The evaluation using historical power emergency events. By extracting event keywords via text mining and identifying the most relevant knowledge base matches via case similarity analysis, this yields optimal equipment/supplier recommendations. We validate superiority by comparing our multi-agent approach with conventional knowledge graph methods. Results demonstrate our method's advantages.

We analyzed five representative power emergency events (2024–2025; see Appendix for details). To prevent bias, we first excluded these events' data from similarity assessments. For each event description, we conducted platform-compatible word segmentation and vectorization. Table 2 displays representative word vectors from Case 1.

**Table 2:** Keyword vectors for case studies (case 1, partial data).

**Keywords**	**Feature vector**
Blizzard	[8.3786594e-03 −5.2053598e-04 −9.4238138e-03 4.7852471e-03 −6.0076267e-03 6.5374356e-03 5.4355143e-03 −4.8469724e-03 2.5291601e-03 5.2344715e-03 −3.6079709e-03 −1.6198035e-03 ……]
December	[0.00595575 0.00378833 0.00177153 0.00062343 0.00883377 0.00482771 −0.0077223 0.00798897 −0.00535822 −0.0008678 −0.00930842 −0.00937253 −0.00380538 0.00535653 0.00163865 −0.00082083 −0.00587822 0.00564618 ……]
Power	[−0.00223986 0.00754375 −0.00309575 0.00352622 0.00015496 0.00145419 −0.0003963 −0.00851374 0.0088014 0.00691093 −0.00956929 −0.00714341 0.00212362 0.00022632−0.00280066 0.00870843 −0.0087514 −0.00285309 ……]
Imaging	[5.4290597e-03 6.8533416e-03 6.1071278e-03 −9.5195500e-03 9.6691269e-03 −8.0433693e-03 6.9033791e-04 8.7057626e-05 7.1318117e-03 −8.8781007e-03 3.1964756e-03 −6.1212792e-03 ……]
Cut	[9.6368423e-04 8.6120395e-03 −4.0091206e-03 2.9682850e-03 3.2174194e-03 −6.5832688e-03 9.1116382e-03 5.5184015e-03 4.8041744e-03 −1.6249869e-03 9.6991528e-03 3.9087841e-03 ……]

We computed case-to-knowledge base similarity scores using vector distance metrics. The highest-scoring match yielded the optimal equipment supplier. Table 3 presents the recommended supplier (with database-derived identifiers). These recommendations help purchasers overcome emergency equipment procurement challenges and which proves particularly valuable for unprecedented events.

**Table 3:** Recommended optimal equipment and suppliers for case study (to be procured).

**Cases**	**Emergency equipment**	**Optimal equipment supplier**
1	Engine powered winch	PJM-9: Hangzhou Yongchuang Machinery Co., Ltd
	Grinding rope	PMS-25: Jiangsu Langshan Steel Wire Rope Co., Ltd
	Glare flashlight	PQG-7: Shenzhen Fenix Lighting Technology Co., Ltd
2	Pay-off rack	PFX-1: Jiangsu Rutong Petro-Machinery Co., Ltd.
	Armor clamp	PJJ-35: Zhejiang Jinlihua Electric Co., Ltd
3	Electric generator	PFF-1: Weichai Power Co., Ltd
	Emergency light	PYJ-1: Ocean's King Lighting Science & Technology Co., Ltd.
	Emergency water pump	PYS-23: Shanghai Jindun Fire-fighting Security Equipment Co., Ltd.
4	Insulation resistance tester	PJY-2: Sieyuan Electric Co., Ltd.
	Multimeter	PWY-16: Xi'an Shengli Instrument Co., Ltd.
5	Infrared thermometer	PWY-16: Xi'an Shengli Instrument Co., Ltd.
	Deicing robot	PCB-10: State Grid Intelligence Technology Co., Ltd.

#### 4.5.2 Comparison of model advantages

Current research predominantly employs knowledge graphs as foundational frameworks for decision-support systems. We accordingly evaluate our model's effectiveness through comparative analysis with established knowledge graph approaches proposed by Sun et al. (2023), highlighting our methodological advantages. For all five case events, we compared each model's recommended supplier counts against actual procurement data. Table 4 presents these comparative results. Our model's recommendations demonstrate significantly closer alignment with actual procurement outcomes than the baseline knowledge graph approach. These results validate our model's superior efficacy.

**Table 4:** Comparison of different models with actual procurement data.

**Case**	**Emergency equipment**	**Optimal equipment supplier (supplier number)**	**Knowledge graph model recommendation (supplier number)**	**Supplier of actual procurement**
1	Engine powered winch	PJM-9	PJM-2	PJM-9
	Grinding rope	PMS-25	PMS-33	PMS-24
	Glare flashlight	PQG-7	PQG-15	PQG-7
2	Pay-off rack	PFX-1	PFX-13	PFX-1
	Armor Clamp	PJJ-35	PJJ-1	PJJ-2
3	Electric generator	PFF-1	PFF-23	PFF-1
	Emergency light	PYJ-1	PYJ-6	PYJ-1
	Emergency water pump	PYS-23	PYS-23	PYS-23
4	Insulation resistance tester	PJY-2	PJY-9	PJY-4
	Multimeter	PWY-16	PWY-12	PWY-16
5	Infrared thermometer	PWY-16	PWY-7	PWY-16
	Deicing robot	PCB-10	PCB-45	PCB-2

## 5 Summary

This study constructs a 4D relationship graph modeling power emergency supply chains by employing knowledge engineering and AI techniques to aggregate internal and external emergency supply data from the power industry, tailored to demand patterns. This framework reveals implicit time-event-supplier-equipment correlations, enabling intelligent emergency supply decisions. Our multi-agent supply network emphasizes critical supply chain interdependencies. The system adapts to stakeholder-specific needs, enables holistic data analysis, and enhances both data utility and emergency decision-making.

Leveraging digital technologies (data processing, mining, and algorithmic modeling), we develop a conceptual framework for power emergency supply decision support. The four-tier architecture (data, fusion, reasoning, application) enables a complete data-to-knowledge-to-service pipeline. This system delivers multidimensional knowledge guidance and optimized decision pathways for emergency supply management.

## 6 Research limitations and future directions

While this conceptual framework establishes foundational planning, several limitations require attention. First, internal data (event records, policies) demonstrate high reliability, but external supplier data necessitates advanced validation techniques to ensure accuracy. Second, internal data interoperability and sharing protocols require refinement. Additionally, external data acquisition demands improved timeliness and quality control. Furthermore, knowledge base construction methodologies need comprehensive development. Finally, the platform requires modular, scalable architecture for decision-support services. It is imperative to conduct high-quality learning and training on extensive samples of knowledge reasoning models, accurately comprehend the interrelationships between events, and precisely investigate and assess the evolutionary patterns and developmental trends of such events. The challenge in enhancing the knowledge service platform lies in effectively leveraging graph analysis technology to optimize data resource utilization, enabling intelligent cognition and reasoning. Additionally, it involves integrating human-computer interaction to accurately transmit and provide feedback on reasoning requirements, as well as consolidating reasoning results for decision-making assistance.

## Data Availability

The original contributions presented in the study are included in the article/supplementary material, further inquiries can be directed to the corresponding author.

## Appendix

**Case Summaries:**

**Case 1:**

On February 27, the Jinhua region was hit by a cold wave, causing the overhead ground wire of the 220 kV Xizhai 4400 transmission line to detach due to severe ice accumulation, resulting in a power outage.

**Case 2:**

On June 25, due to flood discharge from the Xin'anjiang Reservoir, the water level of the Lan River continued to rise, reaching the warning level of 28 meters. Five major reservoirs in Lanxi were near full capacity, and the Jinshantou Reservoir began discharging water.

**Case 3:**

As Typhoon In-Fa approached, to ensure power supply resilience, State Grid Yiwu Power Supply Company canceled all weekend leave for its employees starting July 24, mobilizing full efforts for typhoon response. Over 10 emergency repair teams were formed, with 120 repair personnel, 80 service vehicles, and 15 backup generators deployed. Emergency staff maintained 24/7 readiness to address contingencies.

**Case 4:**

On the morning of April 7, the Integrated Energy Team of State Grid Lanxi Power Supply Company received an urgent repair request from Sanjiang Warehouse. The manager reported a power failure on a docked vessel at the Nübu Port. The team promptly responded, conducting a comprehensive inspection of wiring and sockets using multimeters and insulation resistance testers to ensure stable power restoration.

**Case 5:**

On February 5, a renewed cold wave swept through Zhejiang, triggering a sharp temperature drop and prolonged, widespread rain and snow. The extreme weather caused severe ice accumulation on high-altitude transmission lines, posing critical risks to grid stability. According to State Grid Jinhua Power Supply Company's Transmission Monitoring Center, multiple lines in Jinhua faced hazardous ice buildup.
